# Towards Universal Screening for Colon Cancer: A Cheap, Reliable, Noninvasive Test Using Gene Expression Analysis of Rectal Swabs

**DOI:** 10.5402/2012/170210

**Published:** 2012-01-29

**Authors:** Andrew P. Smith, Yanek S. Y. Chiu, Nancy M. Lee

**Affiliations:** California Pacific Medical Center Research Institute, San Francisco, CA 49107, USA

## Abstract

Though colon cancer is the second leading cause of cancer deaths in the US, it is entirely preventable through early screening to detect and remove adenomatous polyps. Colonoscopy has long been regarded as the “gold standard” but is expensive, invasive, and uncomfortable, and only about half those considered at risk for colon cancer currently submit to colonoscopy or to less reliable alternatives such as fecal occult blood test. Here we describe the use of gene expression analysis to detect altered expression of certain genes associated with not only colon cancer but also polyps. The analysis can be performed on rectal swabs, with specimens provided in a routine doctor's office visit. The existence of this cheap and simple test, together with an active program to encourage individuals to submit to screening, could help eradicate colon cancer.

## 1. Introduction

Colorectal cancer is the third most common cancer in the US and continues to be the second leading cause of cancer deaths. Each year, about 150,000 new cases will be diagnosed and 50,000 patients will die of this disease. These data have not changed much despite increased efforts over the last decade to persuade individuals at risk to undergo screening. In the US, colonoscopy remains the so-called gold standard, because it can detect not only early colorectal cancer but also significant precancerous polyps such as the serrated adenomas or the villotubular adenomas, which can be removed by the same procedure. In principle, then, colon cancer is entirely preventable.

Colonoscopy is expensive and invasive, however, and requires a colon preparation prior to surgery. Individuals without health insurance, and of lower socioeconomic status, are less likely to have a colonoscopy. Some of these individuals opt for other recommended screening procedures, including combination of fecal occult test for blood in the stool annually or flexible sigmoidoscopy every 5 years. These tests are also more common in many countries outside the US, due to financial limitations. Lately, with the US in recession and major cutbacks proposed on health care expenditures, even expert gastroenterologists have suggested that perhaps colonoscopy should not be the gold standard for colon cancer screening [1]. In any case, currently only about 55% of recommended individuals, including all individuals over 50 years old, are screened by any procedure.

In February of 2010, the National Institutes of Health (NIH) organized a Consensus Conference, bringing together with a public representative a group of experts representing the fields of cancer surveillance, health services research, community-based research, informed decision-making, access to care, healthcare policy, health communication, health economics, health disparities, epidemiology, statistics, thoracic radiology, internal medicine, gastroenterology, public health, end-of-life care [2]. The conclusions at this Consensus Conference will have far-reaching consequences on how the US will try to cope with the challenges of early detection of colorectal cancer with improved 5-year survival rates. Noting that the low screening rate in this country is the major obstacle to prevention of colon cancer, the Conference suggested the following ways to improve this rate:

1. eliminate financial barriers to colonoscopy;
2. promote interventions that have been shown to be effective in persuading people to be screened;
3. develop more effective educational programs for targeted patient groups, such as those who lack health insurance and/or are of lower socioeconomic status;
4. implement approaches that will ensure appropriate followup of positive screening results;
5. develop systems to ensure the high quality of colorectal cancer screening programs;
6. conduct studies to determine the comparative effectiveness of the various colorectal cancer screening methods currently in use.

The development of a cheaper and less invasive screening procedure that could detect both cancer and precancerous polyps as reliably as colonoscopy would address most of these issues. In particular, a cheap, simple, noninvasive test that could be performed by any physician would remove the financial and emotional barriers to screening. Several possible alternatives are currently being researched, including and stool tests for DNA mutations associated with colon cancer [3, 4] and various types of blood tests for colon cancer markers [5]. The capsule camera approach, while relatively expensive and also requiring a colon prep, is another less invasive alternative to colonoscopy [6]. While all of these approaches show some promise, none of them to date has achieved a degree of sensitivity or specificity equal to that of colonoscopy.

In this paper, we will discuss our research to develop a rectal swab test, without the need for a bowel preparation. Those individuals testing positive will be recommended for immediate diagnostic colonoscopy to remove any large precancerous polyps or resect an early cancer. Individuals repeatedly testing negative in the swab test should not require colonoscopy. Our aim is that the high sensitivity and specificity of this test will compare favorably with all existing screening modalities.

## 2. Materials and Methods

### 2.1. Animal and Human Tissues

Tissues examined in our studies included the entire colon of mouse (C57BL/6J-min/+ mice, and the wildtype littermates were obtained from Jackson Laboratory, Bar Harbor, ME, USA), which was removed from the animals, opened longitudinally, and washed in cold phosphate-buffered saline, polyps and morphologically normal colon tissue removed from patients undergoing colonoscopy at the California Pacific Medical Center (CPMC), and colon cancer removed from patients undergoing surgical resection at CPMC. The appropriate procedure for obtaining formed consent was followed for all individuals participating in these studies. All samples from human patients were snap-frozen on dry ice as soon as possible within 30 minutes of surgery, then taken immediately to the laboratory for RNA preparation (see below).

### 2.2. Extraction and Preparation of RNA

Total RNA was extracted from tissues using RNAeasy kits from Qiagen (Valencia, California). RNA samples were treated with RNase-free DNase to remove any genomic DNA contamination and were reverse-transcribed. Fifty ng of cDNA from each sample were used as template for PCR amplification with specific oligonucleotide primers using the Applied Biosystems 5700 Sequence Detection System (PE Applied Biosystems, Foster City, California). PCR reactions were performed according to the manufacturer's instructions using the SYBR Green PCR Core Kit (PE Applied Biosystems, Foster City, California).

### 2.3. Analysis of Gene Expression

We analyzed fifteen genes, all of which have previously been shown to be altered in expression in human colon cancer. They fall into four groups, including those involved in the (1) APC/*β*-catenin pathway, including c-myc, cyclin D1, and proliferating peroxisome activating receptor (PPAR*α*) [7, 8]; (2) NF-*κ*B/inflammation pathway, including growth-related oncogene (Gro-*α*), osteopontin (OPN), and colony-stimulating factor (M-CSF-1) [9], cyclooxygenase (COX-)1 and 2, Gro-*γ* (or its mouse homolog, macrophage inflammatory protein-2 (MIP-2)), interleukin-8 (IL-8) (or its mouse homolog, stroma-derived factor (SDF-1)), and the cytokine receptor CXCR2; (3) cell cycle/transcription factor, including p21^cip/waf1^, cyclin D1, c-myc, PPAR*α*, *δ*, *γ* [10, 11]; (4) cell communication signals, including IL-8, PPAR*α*, *δ*, *γ* CXCR2, CD44, and OPN. Most of these genes have been reported to be upregulated in human colon cancers, though some, such as the p21^cip/waf1^, are downregulated. 

Specific primers against each gene were designed using the Primer Express Software (PE Applied Biosystems, Foster City, California). Primer length was 21–27 nucleotides, with a theoretical *T_m_* of 58–60°C. The amplicon size ranged from 66 to 150 bp. Primers were designed to amplify only cDNA template but not genomic DNA template when possible. The specificities of the primers used were demonstrated by the appearance of a single product on 10% polyacrylamide gel electrophoresis and a single dissociation curve of the PCR product.

All the cDNA samples were tested for genomic DNA contamination by using primers for *β*-actin genomic DNA. Using these primers, PCR products derived from the genomic DNA have a different *T_m_* and length from the PCR product derived from cDNA. Only cDNA samples without genomic DNA contamination were used.

 For quantitation of gene expression, the fluorescence of the SYBR Green dye bound to the PCR products was measured after each cycle and the cycle numbers were recorded when the accumulated signals crossed an arbitrary threshold (*C_T_* value). In order to normalize this value, a Δ*C_T_* value was determined as the difference between the *C_T_* value for each gene and the *C_T_* value for *β*-actin, which was determined in each experiment and shown not to vary significantly under the different experimental conditions used in this study. For each gene, a ΔΔ*C_T_* value was determined as the difference between the Δ*C_T_* value for each individual sample and the average Δ*C_T_* value for this gene obtained from the control (wildtype) samples. These ΔΔ*C_T_* values were then used to calculate relative gene expression values as described (Applied Biosystems, User Bulletin no. 2, December 11, 1997).

### 2.4. Statistical Analysis

We used the Wilks lambda criterion for a multivariate analysis of variance (MANOVA) to compare the patterns of expression levels of several genes from cancer versus normal subjects. This test takes into account correlations among gene expression levels and controls the false positive rate by testing the global hypothesis of no differences in gene expressions between cancer and normal subjects. If the test was significant; that is, there was evidence that expression patterns differ, then we used univariate *t*-tests to determine which genes were contributing to the global difference and which were not. All statistical tests were carried out on log (base 2) of the gene expression data since this transformation is required to achieve normal distribution of values.

In some studies, we also determined the Mahalanobis distance (M-dist). This measure summarizes, in a single number, the differences in a pattern of gene expression, for any individual against the average of a pool of individuals, taking into account variability of each gene's expression and correlations among pairs of genes. It is thus well suited both for comparing a control population with an experimental group, such as individuals with cancer, as well as determining the degree of similarity or fit that an individual of unknown characteristics has to a well-characterized group. The latter is what allows M-dist to be used to determine how significantly an individual value differs from a group of controls, which is necessary in screening.

 To perform the calculations, first, for each control biopsy (total of 105), we calculated its M-dist from the multivariate mean of the other 104 control biopsies. We plotted ordered M-dist for the 105 control biopsies against the theoretic expected order statistics for the appropriate chi-squared distribution, to verify that control gene expression values (log base 2) were multivariate normal. Then we computed an M-dist for the gene expression data for each biopsy from each individual with polyps, where M-dist measured the individual's multivariate distance (i.e., difference in pattern of expression) from the pooled mean of the 105 control biopsies.

 M-dist can be converted to *P* values by reference to a chi-squared distribution with degrees of freedom equal to the number of variables (i.e., genes). Using this approach, one can determine an upper bound for the normals, at any arbitrary level of significance, such as the 95th or 99th percentile. This allows analysis of significance of gene expression values of any individual experimental subject as compared to the pool of controls.

## 3. Results and Discussion

Gene expression changes in colon mucosa of a mouse model of colon cancer. We began our studies by examining the APC^min^ mouse [12]. These animals are engineered to contain a mutant form of the human gene adenomatous polyposis coli (APC). As in humans with this mutant gene, these mice develop numerous intestinal polyps at a relatively young age; some of which will progress to locally invasive carcinomas [13]. We first removed polyps from these animals and analyzed them for expression of fifteen genes.

When we analyzed polyps that were removed from these animals at various ages, we observed a wide range of expression levels of these genes, ranging from several that were dramatically upregulated to several that were modestly upregulated, others that exhibited no significant change in expression level, and several that were downregulated. As shown in Table 1, five genes—COX-2, GRO-*α*, CXCR2, OPN, and MIP-2—exhibited a particularly high degree of altered expression in adenomatous polyps (*P* < 0.001). All of these genes have also been reported to be upregulated in human colon cancer or other cancers, though not to such a high degree [11, 14–16].

In studies like this that have been carried out previously by other investigators, it has been assumed that gene expression values in normal appearing mucosa in the mutant mice, in regions away from the polyp, would be similar to those in control mice without polyps. However, when we actually compared the two, we found it was not the case. In these experiments, polyps were removed from the intestines of APC^min^ mice at three different ages—6, 13, and 23 weeks old—and the polyp-free intestines compared with normal colon tissue from wildtype littermates. The intestines were divided into six equal segments of approximately 1.5 cm in length, colonic mucosa was isolated, and the expression of the five genes most altered in polyps analyzed.

**Figure 1:**
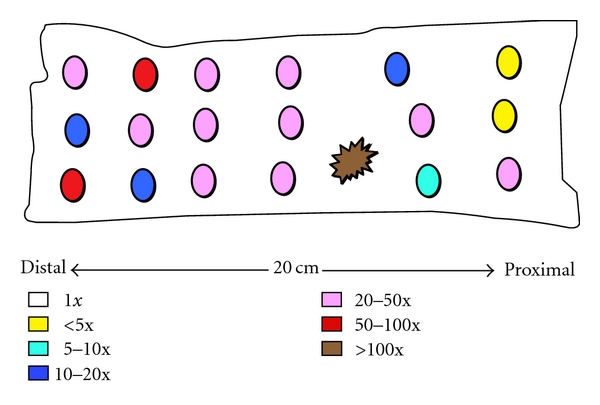
Distribution of IL-8 expression in sigmoidal-rectal colon of a single cancer patient. The patient had a cancer in the sigmoidal-rectal colon, as indicated by the spot with jagged edges. Locations of other mucosa samples removed for analysis are indicated by the circular spots. Levels of IL-8 expression in each sample were determined by RT-PCR and are indicated roughly by color coding, as shown in the Figure. The mean level of expression of IL-8 in colon mucosa of noncancer patients was 2.25 (as shown in Table 3).

While the expression levels of a particular gene in a particular segment at a particular age showed little variability from one wildtype animal to another, there was considerable variation in values for APC^min^ mice. As shown in Table 2, all of these genes except OPN were significantly upregulated, relative to wildtype mucosa, in at least some segments. Of particular interest was that the distribution of these metabolic alterations was not correlated with the presence of polyp (data not shown, but see Figure 1, below); that is, the greatest differences in expression were not necessarily in a region that had been close to the location of a polyp. This finding suggests that the observed changes in gene expression were not the result of a field effect, caused, for example, by escape of altered cells from the polyps, but were intrinsic to the morphologically normal cells of the colon where they were detected.

### 3.1. Gene Expression Changes in Cancer and Normal Mucosa of Human Patients

We next analyzed colon samples from human patients who had previously undergone surgery to remove colon carcinomas [8]. As noted earlier, all the genes we analyzed in mouse have been shown by other laboratories to be differently regulated (up or down) in human colon cancers. Such studies, however, have generally assumed that morphologically normal colon mucosa adjacent to the tumor is metabolically normal and, indeed, have used such tissue as a baseline for comparison. Because of our findings with APC^min^ mice, we were interested in determining whether this assumption is actually valid or whether altered gene expression profiles exist even in morphologically normal colon of cancer patients. For this study, we thus compared gene expression levels in morphologically normal appearing colon mucosa from cancer patients with levels in mucosa from noncancer patients. In both cases, the normal appearing mucosa was removed as biopsies during a colonoscopy. We analyzed two sets of data, one set consisting of samples from patients with cancer in the sigmoidal-rectal region and the other samples from patients with cancer in the ascending colon region. In both studies, we examined expression levels of the same fifteen genes that were analyzed in APC^min^ mice, except for Gro-*γ*, the human analog of MIP-2 in mouse, and IL-8, a close relative of SDF-1 in mouse.

As with the mice, we observed great variability of expression levels in morphologically normal mucosa from cancer patients (Table 3). However, expression levels for several genes tended to be much higher for some samples from cancer patients than for any colon mucosal samples from noncancer patients. For example, four of the genes that were significantly upregulated in normal appearing mucosa of APC^min^ mice—CXCR2, GRO-*α*, COX-2 and OPN—were upregulated in normal appearing mucosa of some cancer patients to levels of 50–200 times relative to that of most values in noncancer patients. In addition, in some cancer patients, PPAR *α*, *δ*, and *γ* were downregulated fifty to one hundred times relative to normal colon mucosal biopsies from noncancer patients. 

All together, seven genes appeared to be significantly upregulated in morphologically normal mucosa of sigmoidal-rectal cancer patients, relative to mucosa of noncancer patients: M-CSF-1, OPN, IL-8, COX-2, CXCR2, p21, and CD44. An additional two genes—PPAR *δ* and *γ*—were shown to be significantly downregulated (Table 3). Quite similar results were obtained for ascending colon. Six of the seven genes significantly upregulated in sigmoidal-rectal mucosa were also upregulated in ascending colon—M-CSF-1, OPN, IL-8, COX-2, CXCR2, and CD44—along with COX-1. Likewise, the same two genes, PPAR *δ* and *γ*, were significantly downregulated in expression in sigmoidal-rectal colon and were also downregulated in ascending colon (Table 3). 

The samples of normal-appearing mucosa from cancer patients that were analyzed for the data in Table 3 were taken from all areas of the surgical section. Figure 1 shows schematically the distribution of samples from a single cancer patient and indicates the approximate expression level in each sample of a single gene, IL-8. It can be seen that there was no correlation of expression level with distance from the cancer, just as there was no correlation of expression level with distance from polyp in APC^min^ mice. A relatively high level of IL-8 expression might be found distant from the tumor, while a low level might be found closer to the tumor. Similar results were obtained with other differently regulated genes. 

These observations strongly suggest that the differently regulated areas of gene expression in normal-appearing colon mucosa of cancer patients did not result from a field effect of spreading cells from the original cancer. It appears that, in individuals with cancer, the normal-appearing colon mucosa has developed abnormalities that can be detected at the molecular level. Polley et al. [17] have confirmed the existence of similar changes using protein expression. For example, they reported changes in expression of more than two hundred different proteins when mucosa of individuals with no polyps were compared with mucosa of individuals with polyps. Subsequent studies studying methylation patterns of several genes in mucosa found differences associated with both aging and the development of carcinogenesis [18, 19], which could be critical steps in the conversion of normal mucosa to polyps and cancer [20]. It is also relevant to note that a study by Øgreid and Hamre [21] reported the presence of mutations in k-ras in stool of a patient eighteen months before the appearance of a malignant polyp. Clearly there are molecular changes occurring in the colon long before the appearance of malignancies. 

To summarize these studies, morphologically normal colon mucosa in APC^min^ mice and in human cancer patients is not metabolically normal. Altered gene expression in this tissue does not appear to result from a field effect, because there was no correlation between extent of altered regulation and distance from polyp or tumor. Our data suggest that alterations of expression levels of certain genes may be an early event in carcinogenesis and may serve as a marker of risk to development of colon cancer. 

### 3.2. Altered Gene Expression in Individuals with Polyps

We next examined whether these alterations in gene expression patterns could also be observed in morphologically normal colon mucosa of individuals with adenomatous polyps [22]. We analyzed a total of 169 rectosigmoid biopsies from 24 individuals with adenomatous/hyperplastic polyps versus 105 rectosigmoid biopsies from 17 control individuals without polyps. The polyps were located in different regions of the colon, with 6 individuals presenting with a polyp in the transverse region, 7 in the ascending/descending region, and 13 in the rectosigmoid area. All the biopsies of morphologically normal tissue were taken randomly and away from the polyp, though we cannot rule out the possibility that, in patients with a polyp in the rectosigmoid region, this polyp had some effect on metabolism in normal-appearing surrounding tissue. Eight of the twenty-four patients with polyps were individuals with a relative (first- or other degree) with colon cancer, or with a personal history of colon cancer or of some other form of cancer. However, none of the 17 control individuals had a known family or personal history of cancer. 

To distinguish any effects of personal/family history alone from the presence of polyps, we initially carried out three group-wise comparisons: (1) individuals with polyps and no personal/family history versus controls, (2) individuals with polyps and personal/family history versus controls, and (3) individuals with polyps and personal/family history versus individuals with polyps and no history. The first two comparisons, individuals with polyps and with or without history versus controls, were significant, whereas there was no significant difference in gene expression levels between individuals with history and without history. 

Further analysis was carried out on individual biopsies, using the Mahalanobis measure. We compared the M-dist for controls and for individuals with polyps, plotted on a logarithmic scale. A log-rank test comparing the distribution of all biopsies from individuals with polyps versus all controls indicated a highly significant difference (*P* < 0.001). Moreover, gene expression values above an M-dist value of 25 corresponded to the 95th percentile; that is, all values above this cutoff were significantly (*P* < 0.05) higher than the pooled mean of gene expression values of all control biopsies. We found that 20/24 individuals with polyps had at least one biopsy with a gene expression value above this cut-off point (and 17 had two or more biopsies fulfilling this criterion) versus 5/17 controls with one or more biopsies with a gene expression value above the cutoff (and just one control with two biopsies meeting this criterion). 

To summarize, this study found that normal-appearing colon in individuals with polyps, like that we had previously demonstrated in individuals with colon cancer, exhibited altered levels of gene expression. Thus these changes occur relatively early in the carcinogenetic process, before the appearance of an actual cancer. 

### 3.3. Altered Gene Expression in Individuals with a Family History of Cancer

Since our previous studies had indicated that the presence of either adenomatous polyps or colon cancer in humans is associated with significant alterations in the expression of certain genes in the normal-appearing portion of the colon, we next examined whether such changes exist even in individuals with no polyps but possibly at risk for cancer by virtue of a family history of the disease [23]. We employed the same gene panel as in our previous studies, except for the presence of an additional gene, serum amyloid A1 (SAA1). 

Twelve individuals with a family history of colon cancer in a first-degree relative and sixteen individuals with no known family history of colon cancer were included in the study. Biopsy samples of normal-appearing colon mucosa were obtained from the ascending, transverse, descending, and rectosigmoid regions of the colon (2–8 biopsy samples were obtained from each region). Relative to normal controls, the expression of several genes, including PPAR-*γ*, SAA1, and IL-8 were significantly altered in the macroscopically normal rectosigmoid mucosa from individuals with a family history of colon cancer. Thus molecular abnormalities that precede the appearance of adenomatous polyp are present in the mucosa of individuals who have a family history of colon cancer. This observation underscores the importance of screening for individuals with family history of cancer, as well as suggests the usefulness of this screen for individuals who may be uncertain of their family history. 

Multivariate analysis of the expression values of all sixteen genes indicated a significant difference in the biopsy samples from the rectosigmoid region (*P* = 0.01) between those with and those without a family history of sporadic colon cancer. Gene expression in biopsy samples from the descending, ascending, and transverse colon did not vary significantly between these two groups of individuals (*P* = 0.06, 0.22, and 0.52, resp.). Most of the differences in rectosigmoid biopsy samples were contributed by just five of these genes: PPAR-*γ*, SAA1, IL-8, COX-2, and PPAR-*δ*. Similar to the alterations of gene expression in the normal colon mucosa of cancer patients, we found that the expression levels of IL-8 and COX-2 were upregulated, and those of PPAR-*γ* and PPAR-*δ* were downregulated in the mucosa of individuals with a family history of sporadic colon cancer. 

### 3.4. Analysis of Gene Expression Using Rectal Swabs

The studies discussed above demonstrate that morphologically normal colon mucosa from individuals with colon cancer or at increased risk for colon cancer have altered gene expression patterns, which could be the basis for screening. However, all of the studies we have discussed to date involved removal of biopsies during colonoscopy. Since the point of developing a molecular screening process is to avoid the necessity of colonoscopy, we next sought to develop a more noninvasive way of obtaining colon mucosa samples. Using an anoscope, we inserted a soft brush about 2 cm. into the colons of individuals and gently swabbed to remove colon mucosal cells.

These cells were removed from the brush by dipping and swirling it in a buffer. This was followed by extraction of RNA, preparation of cDNA, and PCR. In this manner, we compared rectal swabs with biopsies from 90 patients, who included individuals with no polyps, but with family or personal history of cancer, individuals with adenomatous polyps (with or without history), control individuals with neither history nor polyps or colon cancers, and cancer patients.

Analysis of individuals with cancer, polyps, or family/self-history of cancer clearly showed that gene expression profiles of swab samples were very similar to profiles of samples obtained by biopsies (Table 4). All these groups of individuals were significantly different from a control group without history or polyps when expression of the entire panel of sixteen genes was analyzed multivariately. Moreover, a large majority of individuals, ranging from about 70 to 84%, exhibited significantly different expression values of swabs compared to the pooled controls (Table 5). 

 Biopsy data showed comparable numbers (68–80%). These data indicate that the sensitivity of our gene expression analysis to detect individuals of cancer risk is quite high if multiple rectal swabs are analyzed. Some patients in our polyps' group also had family history or self-history but no subject in the family/self-history (FHSH) group had polyps. This may have resulted in a higher percentage of significantly different individuals in the polyps group than in the FHSH group. 

The cancer group was very small, consisting of just five individuals. But gene expression analysis indicated that not only did the cancer group differ significantly from the control group but each of five individuals was highly significantly different from controls as well. The M-dist values of 48 out of 50 (96%) of our total swab samples from these five individuals were above the 95th percentile line. This suggests a high sensitivity of this assay to identify individuals with colon cancer, higher than has generally been reported using stool analysis of gene mutations [4]. While this is a very small number of subjects and will require studies with larger patient pools, we reported similar results with seven additional colon cancer patients in our earlier studies using normal-appearing mucosal tissue taken from the margins of resected colon cancer [8]. 

Furthermore, in one case, swabs were taken from an individual with cancer both before as well as after bowel preparation. The altered gene expression profile was highly significant in both instances. While further studies will be required to support this conclusion, this result suggests that the rectal swab procedure may be able to dispense with bowel preparation. This is another significant disadvantage associated with colonoscopy that undoubtedly contributes to poor patient compliance, so eliminating it should further increase the attractiveness of the swab procedure. 

## 4. Conclusions 

Our studies suggest that gene expression analysis may be suitable as a screening process to identify individuals at risk for developing colon cancer. While promising advances have been made in the use of both DNA stool tests [3, 4] and blood-borne biomarkers [5], none of these tests has yet shown it is capable of equaling colonoscopy for sensitivity and specificity, so different approaches should continue. The use of rectal swabs is a noninvasive procedure that can be carried out in any doctor's office and perhaps also at home by individuals with a properly prepared kit as is currently used for fecal occult blood analysis. Moreover, Ahmed et al. [24] were able to isolate RNA from stool samples and, using an RT-PCR technique similar to ours, were able to distinguish polyps and more advanced stages of colon cancer from each other as well as from controls, in some cases by expression of a single gene. Gene expression analysis can also be used in certain blood tests for colon cancer [5]. Very recent work in our own laboratory has suggested that these changes in gene expression may also be detected using buccal (cheek) swabs, a still easier and less invasive procedure and that the technique may be applicable for detecting other diseases associated with the gastrointestinal tract (Lee et al. “unpublished data”). So gene expression analysis may have a wide spectrum of applications for cancer screening. 

As applied to colon cancer, as we envision it, gene expression would not replace colonoscopy but allow its limited resources to be focused on those individuals whom expression analysis indicates are most likely to have polyps. Given the large and growing variety of tests being explored, it may be that a combination of more than one type of test will prove to have the highest sensitivity to detection of cancer and polyps. If individuals who are free of polyps and cancer can be reliably identified without colonoscopy, it would result in an enormous reduction of needed resources, for both individuals and society. 

## Figures and Tables

**Table 1:** Relative gene expression levels in colon polyps of APC^min^ mice (mean ± SE).

No.	Gene	Wildtype littermate	Individual polyp	*P* value
1	OPN	1.62 ± 0.60	430.38 ± 125.24	<0.01
2	MIP-2	1.74 ± 1.60	202.74 ± 43.40	<0.001
3	Gro-*α*	1.40 ± 0.32	122.48 ± 18.97	<0.001
4	CXCR2	1.41 ± 0.35	104.51 ± 23.31	<0.001
5	COX-2	1.41 ± 0.25	81.64 ± 16.36	<0.001
6	Cyclin D1	1.34 ± 0.34	19.48 ± 2.67	<0.001
7	SDF-1	1.23 ± 0.34	11.02 ± 2.45	<0.01
8	c-myc	1.09 ± 0.18	6.49 ± 0.96	<0.001
9	M-CSF1	1.05 ± 0.15	4.26 ± 1.60	NS
10	CD44V6	1.17 ± 0.28	3.78 ± 0.61	<0.01
11	COX-1	1.07 ± 0.15	3.24 ± 0.60	<0.01
12	PPAR-*γ*	1.13 ± 0.22	0.86 ± 0.24	NS
13	p21^cip/waf1^	1.11 ± 0.17	0.51 ± 0.07	<0.05
14	PPAR-*δ*	1.16 ± 0.27	0.44 ± 0.05	<0.05
15	PPAR-*α*	1.04 ± 0.12	0.17 ± 0.03	<0.001

Gene expression levels were determined using RT-PCR. In no. 1~5, *n* = 13 in the wildtype littermate group; *n* = 14, in the individual polyp group; in no. 6~15, *n* = 6 in the wildtype littermate group; *n* = 10 in the individual polyp group. Significance was determined by *t*-test.

**Table 2:** Multivariate analysis of gene expression in normal-appearing colon mucosa of 23 week old APC^min^ mice, as compared to colon mucosa of normal mice.

Colon segment						
Gene	1	2	3	4	5	6
COX-2	++	++++	+	++++	+	+++
CXCR-2	+	++	—	++	+	++++
MIP-2	++	++	—	++	+	+++
Gro-*α*	—	+	—	—	+	+++
OPN	—	—	—	—	—	—

Colons were removed from animals and any polyps removed. The colons were then divided into 6 segments, colon mucosa isolated, and gene expression determined as described in the Material and Methods section, with values for APC^min^ mice compared to those for wildtype mice. Multivariate analysis was performed on these values as described in the Material and Methods section, in which the significance of the difference in expression between APC^min^ and wildtype mice was determined for each gene in the presence of all the other genes. For this analysis, 6 mice were used for each group (APC^min^ and wildtype), and 1 mucosa sample analyzed per segment per mouse, +: *P* < 0.05; ++: *P* < 0.01; +++: *P* < 0.001; ++++: *P* < 0.0001.

**Table:** (a) Sigmoidal-rectal colon

		Normal subjects	Cancer patients	
	Gene	Mean + SD	Range	*P* value
1	CXCR2	1.30 ± 1.11	0.81–210.11	<0.01
2	Gro-*α*	2.93 ± 6.93	0.78–104.69	NS
3	IL-8	2.25 ± 2.63	1.22–82.14	0.0001
4	COX-2	1.80 ± 2.63	0.91–66.26	0.001
5	OPN	1.55 ± 2.04	0.94–58.08	0.0001
6	Gro-*γ*	1.92 ± 3.34	0.80–36.50	NS
7	M-CSF-1	1.54 ± 1.40	1.54–30.70	0.0001
8	COX-1	1.22 ± 0.87	0.12–9.58	NS
9	CD44	1.12 ± 0.56	0.54–6.52	<0.05
10	c-MYC	1.24 ± 0.82	0.12–4.76	NS
11	Cyclin D	1.28 ± 0.84	0.43–4.44	NS
12	PPAR-*α*	1.10 ± 0.62	0.02–2.87	NS
13	PPAR-*δ*	1.15 + 0.55	0.023–1.90	<0.01
14	P21	1.04 + 0.29	0.40–1.68	<0.01
15	PPAR-*γ*	1.07 + 0.40	0.01–1.28	<0.01

**Table:** (b) Ascending colon

		Normal subjects	Cancer patients	
	Gene	Mean + SD	Range	*P* value

1	CXCR2	1.32 + 1.08	1.90–90.20	<0.05
2	Gro-*α*	1.60 + 2.08	0.46–29.90	NS
3	IL-8	1.66 + 1.62	1.32–182.66	<0.05
4	COX-2	1.84 + 3.04	2.96–152.50	0.0001
5	OPN	1.53 + 1.31	9.24–152.98	0.0001
6	Gro-*γ*	1.40 + 1.41	0.63–11.16	NS
7	M-CSF-1	1.68 + 1.62	4.01–40. 19	0.0001
8	COX-1	1.17 + 0.75	0.84–44.90	<0.001
9	CD44	1.11 + 0.51	0.99–13.63	0.0001
10	c-MYC	1.16 + 0.63	0.39–10.82	NS
11	Cyclin D	1.38 + 1.08	0.12–13.15	NS
12	PPAR-*α*	1.16 + 0.58	0.22–4.09	NS
13	PPAR-*δ*	1.13 + 0.55	0.02–7.08	<0.05
14	p21	1.09 + 0.40	0.04–2.66	NS
15	PPAR-g	1.08 + 0.42	0.01–1.14	0.01

Colon mucosa samples were isolated from (a) the sigmoidal-rectal region of noncancer subjects (78 samples from 12 individuals) and from the adjacent normal mucosa of patients with sigmoidal-rectal cancer (62 samples from 5 patients); or (b) from the ascending region of noncancer subjects (39 samples from 11 individuals) and from the adjacent normal mucosa of patients with ascending colon cancer (65 samples from 4 patients). Samples were analyzed for gene expression as described in the Material and Methods section. Means + standard deviations are given for noncancer subjects; ranges are given for cancer patients. Multivariate analysis was then performed on each gene taken in relation to all the other genes, to determine the significance of the difference between cancer and noncancer individuals. NS, not significant at *P* < 0.05 level.

**Table 4:** Significance of three groups versus controls for gene expression levels.

Comparison of overall group	*P* values	
	Swabs	Biopsies
Cancers versus controls	<0.001	NA
Polyps versus controls	<0.01	<0.01
FHSH versus controls	<0.01	<0.01

**Table 5:** Summary of patients with altered gene expression in three groups versus control group.

	Number of patients with altered gene expression	
	Swab samples	Biopsy samples
Cancer (*n* = 5)	5/5 (100%)	NA
Polyps (*n* = 25)	21/25 (84%)	20/25 (80%)
FHSH (*n* = 37)	26/37 (70%)	25/37 (68%)

## References

[1] Neugut A, Lebwohl B (2011). Commentary: sigmoidoscopy versus colonoscopy. *Journal of the American Medical Association*.

[2] Steinwachs D, Allen JD, Barlow WE (2010). National Institutes of Health state-of-the-science conference statement: enhancing use and quality of colorectal cancer screening. *Annals of Internal Medicine*.

[3] Imperiale TF, Ransohoff DF, Itzkowitz SH, Turnbull BA, Ross ME (2004). Fecal DNA versus fecal occult blood for colorectal-cancer screening in an average-risk population. *New England Journal of Medicine*.

[4] Ahlquist DA, Sargent DJ, Loprinzi CL (2008). Stool DNA and occult blood testing for screen detection of colorectal neoplasia. *Annals of Internal Medicine*.

[5] Hundt S, Haug U, Brenner H (2007). Blood markers for early detection of colorectal cancer: a systematic review. *Cancer Epidemiology Biomarkers and Prevention*.

[6] Sieg A (2011). Colon capsule endoscopy compared with conventional colonoscopy for the detection of colorectal neoplasms. *Expert Review of Medical Devices*.

[7] He TC, Sparks AB, Rago C (1998). Identification of c-MYC as a target of the APC pathway. *Science*.

[8] Koh TJ, Bulitta CJ, Fleming JV, Dockray GJ, Varro A, Wang TC (2000). Gastrin is a target of the *β*-catenin/TCF-4 growth-signaling pathway in a model of intestinal polyposis. *Journal of Clinical Investigation*.

[9] Zhang Z, Dubois RN (2001). Detection of differentially expressed genes in human colon carcinoma cells treated with a selective COX-2 inhibitor. *Oncogene*.

[10] Sherr CJ (2000). The pezcoller lecture: cancer cell cycles revisited. *Cancer Research*.

[11] Notterman DA, Alon U, Sierk AJ, Levine AJ (2001). Transcriptional gene expression profiles of colorectal adenoma, adenocarcinoma, and normal tissue examined by oligonucleotide arrays. *Cancer Research*.

[12] Chen LC, Hao CY, Chiu YSY (2004). Alteration of gene expression in normal-appearing colon mucosa of APC min mice and human cancer patients. *Cancer Research*.

[13] Ieda S, Watatani M, Yoshida T, Kuroda K, Inui H, Yasutomi M (1996). Immunohistochemical analysis of p53 and ras p21 expression in colorectal adenomas and early carcinomas. *Surgery Today*.

[14] Eberhart CE, Coffey RJ, Radhika A, Giardiello FM, Ferrenbach S, Dubois RN (1994). Up-regulation of cyclooxygenase 2 gene expression in human colorectal adenomas and adenocarcinomas. *Gastroenterology*.

[15] Li A, Varney ML, Singh RK (2001). Expression of interleukin 8 and its receptors in human colon carcinoma cells with different metastatic potentials. *Clinical Cancer Research*.

[16] Williams NS, Gaynor RB, Scoggin S (2003). Identification and validation of genes involved in the pathogenesis of colorectal cancer using cDNA microarrays and RNA interference. *Clinical Cancer Research*.

[17] Polley ACJ, Mulholland F, Pin C (2006). Proteomic analysis reveals field-wide changes in protein expression in the morphologically normal mucosa of patients with colorectal neoplasia. *Cancer Research*.

[18] Belshaw NJ, Elliott GO, Foxall RJ (2008). Profiling CpG island field methylation in both morphologically normal and neoplastic human colonic mucosa. *British Journal of Cancer*.

[19] Belshaw NJ, Pal N, Tapp HS (2010). Patterns of DNA methylation in individual colonic crypts reveal aging and cancer-related field defects in the morphologically normal mucosa. *Carcinogenesis*.

[20] Yi JM, Dhir M, Van Neste L (2011). Genomic and epigenomic integration identifies a prognostic signature in colon cancer. *Clinical Cancer Research*.

[21] Øgreid D, Hamre E (2007). Stool DNA analysis detects premorphological colorectal neoplasia: a case report. *European Journal of Gastroenterology and Hepatology*.

[22] Hao CY, Moore DH, Chiu YSY (2005). Altered gene expression in normal colonic mucosa of individuals with polyps of the colon. *Diseases of the Colon and Rectum*.

[23] Hao CY, Moore DH, Wong P, Bennington JL, Lee NM, Chen LC (2005). Alteration of gene expression in macroscopically normal colonic mucosa from individuals with a family history of sporadic colon cancer. *Clinical Cancer Research*.

[24] Ahmed FE, Vos P, IJames S (2007). Transcriptomic molecular markers for screening human colon cancer in stool and tissue. *Cancer Genomics and Proteomics*.

